# Optimization of human umbilical cord blood-derived mesenchymal stem cell isolation and culture methods in serum- and xeno-free conditions

**DOI:** 10.1186/s13287-021-02694-y

**Published:** 2022-01-10

**Authors:** Liem Thanh Nguyen, Nghia Trung Tran, Uyen Thi Trang Than, Minh Quang Nguyen, Anh Minh Tran, Phuong Thi Xuan Do, Thao Thi Chu, Tu Dac Nguyen, Anh Viet Bui, Tien Anh Ngo, Van Thanh Hoang, Nhung Thi My Hoang

**Affiliations:** 1 Vinmec Research Institute of Stem Cell and Gene Technology, Hanoi, Vietnam; 2 College of Health Sciences, VinUniversity, Hanoi, Vietnam; 3 VNU University of Science, Vietnam National University, Hanoi, Vietnam; 4 Graduate School of Analytical Science and Technology (GRAST), Chungnam National University, Daejeon, Korea; 5 Center of Applied sciences, Regenerative medicine, and Advance technologies (CARA), Hanoi, Vietnam

**Keywords:** Umbilical cord blood, Mesenchymal stem cells, Muse cells, Autologous plasma, Angiogenesis, Cancer cells

## Abstract

**Background:**

Although umbilical cord blood (UCB) is identified as a source of mesenchymal stem cells (MSCs) with various advantages, the success in cell isolation is volatile. Therefore, it is necessary to optimize methods of cord blood-derived MSC (UCB-MSC) isolation and culture. In this study, we evaluated the efficiency of UCB-MSC isolation and expansion using different commercially available serum- and xeno-free media and investigated the capacity of autologous serum and plasma as a supplement to support cell proliferation. Additionally, we defined the presence of multilineage-differentiating stress-enduring (Muse) cells in the UCB-MSC population. Functions of UCB-MSC in in vitro angiogenesis processes and anti-cancer were also verified.

**Methods:**

Mononuclear cells were isolated using density gradient separation and cultured in four commercial media kits, as well as four surface coating solutions. UCB-MSCs were characterized and tested on tube formation assay, and co-cultured with SK-MEL cells in a transwell system.

**Results:**

The results showed that only StemMACS™ MSC Expansion Media is more appropriate to isolate and culture UCB-MSCs. The cells exhibited a high cell proliferation rate, CFU forming capability, MSC surface marker expression, trilineage differentiate potential, and chromosome stability. In addition, the culture conditions with autologous serum coating and autologous plasma supplement enhanced cell growth and colony forming. This cell population contained Muse cells at rate of 0.3%. Moreover, UCB-MSCs could induce the tube formation of human umbilical vein endothelial cells and inhibit more than 50% of SK-MEL cell growth.

**Conclusions:**

UCB-MSCs could be high-yield isolated and expanded under serum- and xeno-free conditions by using the StemMACS™ MSC Expansion Media kit. Autologous serum coating and plasma supplement enhanced cell proliferation. These UCB-MSCs had effected the tube formation process and an anti-cancer impact.

## Introduction

Mesenchymal stem cells (MSCs) are adult stem cells, which are self-renewing and multipotent progenitor cells. According to the International Society for Cellular Therapy (ISCT), the criteria to define MSCs are their plastic-adherent ability; the high expression of CD105, CD73, CD90 (≥ 95%), as well as the lack of expression of CD45, CD34, CD14/CD11b, CD79a/CD19, HLA class II (≤ 2%); and the capability of differentiating into adipocyte, osteocyte, and chondrocyte under the proper induction conditions [1]. From the first study on MSCs published in 1966 by Fridenshtein et al. to date, there are thousands of clinical trials conducted with the application of MSCs in the treatment of many kinds of diseases [2].

MSCs can be isolated from a variety of sources, the most relevant of which are bone marrow (BM), adipose tissue (AD), umbilical cord tissue (UC), and umbilical cord blood (UCB). Among them, BM has been the most common source for MSCs that has been widely used in clinical trials [3]. However, the isolation of MSCs from BM and AD is problematic due to invasive collection processes that can cause harm to the donors. Moreover, the significant reduction in BM-MSCs proliferative capacity with age has been shown for bone marrow of patients aged 59–75 compared to patients aged 0–18 [4]. In contrast, UCB is considered most suitable as it is free from ethical complications, and is an easy and non-evasive access as MSC source. Furthermore, UCB-derived MSCs can be extensively maintained in culture, are amenable to large-scale expansion, feature a retardation of senescence, and exhibit more potent anti-inflammatory effects than others [5]. UCB-MSCs were reported to exhibit a similar capacity to that of BM-MSC in terms of multi-lineage differentiation, tolerance for aging, and paracrine activity [6, 7], and appears to be superior even in their capacity for proliferation and clonality [5, 8]. Consequently, UCB-MSCs have been more and more progressively studied as an alternative source for clinical application. There are several studies that report the safety and efficiency of using UCB-MSCs in humans for the treatment diseases such as knee osteoarthritis [9], recessive dystrophic epidermolysis bullosa [10], rheumatoid arthritis [11], atopic dermatitis [12], and articular cartilage defects [13].

Despite well-established advantages and obvious benefits, the use of UCB as a major source for isolating MSCs is somewhat hampered because of its low isolation success rate. There is evidence for suboptimal isolation efficiency and stability that ranges from 15 to 50% [14–16]. The low frequency of colonies forming units (CFU) can be seen as well as a criteria for markedly reduced isolation success, especially observed for full-term CB units compared to pre-term CB units [17]. Therefore, the process of isolation and expansion of UCB-MSCs needs to be optimized. Besides methodical limitations, the utilized culture media is one of the most essential factors in fundamental cell culture which provides basic factors essential for MSC isolation, survival, and development. To date, most of basic media that have been used for isolating and culturing UCB-MSCs are supplemented with fetal bovine serum (FBS) as a source of growth-stimulating factors [5–13, 18]. However, the use of FBS should be limited because of the risk of prion as well as viral transmission or adverse immunological reactions against xenogenic components [19]. Synthetic commercial serum-free media with defined xeno-free components have been used as an alternative and have since been well studied and optimized, although the effectiveness of each media on MSCs during cell isolation and culture varies significantly due to the diverse characteristics of different MSCs populations when isolated from numerous sources [20–22].

Another important characteristic of MSCs is the adhesion ability, so the use of factors that support MSCs adhesion processes contribute to an increased isolation yield as well as cell proliferation. So far, several strategies have been developed for supporting cell attachment. Traditional methods used FBS, a highly enriched supplement that provides a wide range of cell attachment proteins, growth factors, and other important biomolecules. Besides FBS, different xeno-free, protein-based coating agents or modified culture surfaces for MSC culture have also been developed and are currently commercially available [22, 23]. These factors, along with other adhesion-supporting protein components, are assumed to contribute to enhancing cell adhesion and proliferation of MSCs in many research applications [24, 25]. Nevertheless, using FBS as coating sulotion also has limitations in term of risky in transferring animal factors to human [26], and the variation effect on each MSC lines depend on different commercial products.

In this study, we present for the first time the UCB-MSC isolation capacity for the use of different commercially available xeno-free media and surface coating solutions. Surprisingly, only one type of MSC medium kit among four commercial kits used in this study had the ability to isolate UCB-MSCs with an efficiency of 90%. We also optimized the proliferation of UCB-MSCs in the primary and early-stage culture with the addition of autologous plasma and autologous serum-surface coating. UCB-MSCs isolated and cultured by our method expressed that standard MSC markers, exhibited suitable clonality and multilineage-differentiation. Moreover, we have defined the percentage of multilineage-differentiating stress-enduring (Muse) cells in both umbilical cord blood mononuclear cell (UCB-MNC) and UCB-MSC populations. In addition, UCB-MSCs showed the ability to inhibit the growth of skin cancer SK-MEL cells; meanwhile maintaining the stemness of MSCs. Furthermore, our study indicated that UCB-MSC conditioned medium had great effects on angiogenesis in an in vitro model.

## Materials and methods

### Ethics statement

The protocols were reviewed and approved by the Ethics Committee of Vinmec International Hospital (document no. 40/2020/QD-VINMEC), in compliance with the Helsinki Declaration. All methods were performed in accordance with the relevant guidelines and regulations. The donors were fully informed about the purposes of study, and their voluntary consent was obtained in written form.

### Materials

This study was conducted with 45 umbilical cord blood (UCB) samples that met the requirements for cryopreservation at the private cord blood bank at Vinmec International Hospital in Vietnam. All donors were healthy and were confirmed not to carry any of the following viruses: human immunodeficiency virus, hepatitis B virus, hepatitis C virus, cytomegalovirus, and syphilis. The materials were tested for transmissible diseases before blood collection. Among them, 10 UCB units were used for MSC isolation and expansion. The CD34^+^ cell number and percentage of SSEA-3^+^ cells were determined in all 45 UCB samples.

### Autologous serum and plasma preparation

Cord blood units from different donors were centrifuged at 1710×*g* for 10 min at 4 °C. After centrifugation, autologous plasma was collected as much as possible without touching the red layer of blood cells. To prepare autologous serum (AutoSerum), a part of collected plasma was treated with thrombin (Sigma) at a concentration (V/V) of 2 µL thrombin/1 mL plasma for blood clotting. Then the mixture was incubated at 37 °C for 1 h before removing the clot. Next, AutoSerum was heat-inactivated at 56 °C for 30 min, centrifuged at 400×*g* for 15 min at room temperature, and the pellet discarded before use.

The remaining autologous plasma (AutoPlasma) was inactivated by heating at 56 °C for 60 min. The plasma was then centrifuged again at 1710×*g* for 5 min at 4 °C. The supernatant was collected and stored at 4 °C.

### Umbilical cord blood mononuclear cell isolation

Umbilical cord blood mononuclear cells (UCB-MNCs) were purified using Ficoll-Paque (GE Healthcare, USA) and density-gradient centrifugation. The red layer containing blood cells remaining after plasma collection was reconstituted with PBS buffer to the corresponding volume before collecting autologous plasma. Then, the blood suspension was layered on the top of Ficoll, centrifuged for 20 min at 840×*g* with low acceleration and no brake at 4 °C. The buffy coat was collected and then washed with phosphate buffer saline (PBS, Gibco, Grand Island, NY, USA) two times by centrifugation at 280×*g* for 8 min.

### UCB-MSC isolation and expansion

UCB-MNCs were grown in 6-well treated plastic plates (Corning, USA) at a concentration of 0.5 × 10^6^ MNCs/mL in a 37 °C humidified incubator containing 5% CO_2_.

Plates were coated with different coating solutions as indicated below at 37 °C for 30 min before cell seeding. Non-adherent cells were discarded after 48 to 72 h of cell seeding and fresh culture medium was added. The medium was replaced every 3 days. When cells reached 70–80% confluence, they were trypsinized with TrypLE (Gibco, USA) and were subsequently re-seeded.

Culture medium kits used in this experiment were StemMACS™ MSC Expansion Media (StemMACS) (Miltenyi Biotec), MesenCult™-ACF Medium (MesenCult) (STEMCELL Technologies), StemPro® MSC SFM CTS ™ (StemPro) (Gibco) and MSC NutriStem® XF Medium (NutriStem) (Biological Industries). All culture media were prepared according to manufacturer’s instructions. To test the effect of AutoPlasma, culture medium was supplemented with 10% (v/v) of this solution.

Surface coating solutions used were CELLstart™ CTS™ (CELLstart), Nutristem MSC attachment solution (Nutristem Attach.) (Biological Industries), and Autologous Serum (AutoSerum), along with non-coated plates that were used as control. CELLstart™ CTS™ and Nutristem MSC attachment solution were diluted at ratio 1:50 with PBS according to manufacturer’s instructions. In brief, 500 µL each of diluted CELLstart, Nutristem Attach., and AutoSerum were applied onto 6-well culture plates, incubated at 37 °C in a humidified atmosphere of 5% CO_2_ in air for 1–2 h. Any extra coating solutions were discarded before adding the cells.

### Fibroblast colony forming unit (CFU-F) assay and population doubling time determination

In order to determine the frequency of fibroblast colony-forming units (CFU-F) grown in different conditions, UCB-derived MSCs were seeded at a density of 100 cells/ cm^2^ at passage 3. The culture medium was changed every three days. After 2 weeks, the cells were fixed with 100% Ethanol and stained with Giemsa 1X (Sigma) and incubated at room temperature for 10 min before removing Giemsa and washed with deionized water. CFU-F colonies were observed under microscope and determined as the number of colonies per 100 cells at seeding.

To determine the proliferation capacity of MSCs under different conditions, the number of UCB-MSCs was counted and viability testing was performed in each subculture. Population doubling time (PDT) was determined at each passage by using the following formula [5]: PDT = CT/CPDs, where CPDs (cumulative population doublings) = log(N/No) × 3.31; N is the cell number at defined time points; N_0_ is the initial number at cell seeding and CT is the time in culture between two time points.

### Flow cytometry analysis

Immunophenotypic analysis of UCB-MSCs was analyzed by flow cytometry using BD Stemflow™ hMSC Analysis Kit (BD Biosciences – USA) with the following markers: CD90, CD73, and CD105 for positive markers; CD45, CD34, CD11b, CD19, and HLA-DR for negative markers. For Muse cell detection, FITC-conjugated SSEA-3 (BD Biosciences – USA) and PE-conjugated CD105 antibodies (BD Biosciences – USA) were used. Surface marker expression was analyzed using the Navios system and FlowJo software. CD34^+^ cell counting was done by using the Stem Kit Reagent (IM3630; Beckman Coulter) containing CD45-FITC and CD34-PE antibodies.

### Multilineage differentiation

The trilineage differentiation capacity of UCB-MSCs was tested at passage 3. Osteogenesis, adipogenesis, and chondrogenesis were initiated using StemPro™ Osteogenesis Differentiation Kit, StemPro® Adipogenesis Differentiation Kit, StemPro™ Chondrogenesis Differentiation Kit (Gibco, USA), respectively. Differentiated cells were confirmed by Alizarin Red staining, Oil Red O staining, and Alican Blue staining, respectively. All staining solutions were supplied together with the kits. StemMACS MSC Expansion Media was used as un-differentiated control. Cells were cultured in differentiation medium for 14 days before being fixed with 4% paraformaldehyde (PFA) (Sigma Aldrich) and stained as mentioned above.

### Karyotyping analysis

Karyotyping was performed on UCB-MSCs at passage 3. When the cells reached logarithmic phase (60%–80% confluence), cells were incubated with KaryoMax Colcemid (Life Technologies, USA), (1:100 dilution) at 37 °C in a 5% CO_2_ incubator for 30 min. A 2 mL trypsin EDTA 0.25% (Gibco) was added into the flask and incubated at 37 °C for 1 to 2 min to detach cells. Cells were incubated in a hypotonic solution (KCl 0.56%) which had been pre-warmed at 37 °C for 30 min. Subsequently, cells were washed with Carnoy’s solution (3:1 v/v absolute ethanol: glacial acetic acid) for 3 times. Cells were transferred on a clean slide for a metaphase spread and incubated at 60 °C overnight. Cells were stained using G-banding technique. Metaphase cells were captured by MetaSystems (Carl Zeiss) and the karyotype analyzed by Ikaros software. Karyogram is 550 bands according to ISCN (An International System for Human Cytogenomic Nomenclature) 2016 standard.

### Stress condition by long-term trypsin incubation

MSCs at passage 3 were detached by using CTS™ TrypLE™ Select Enzyme (Gibco, USA). And then 10^6^ cells were collected and suspended in 5 mL of TrypLE at 37 °C, 5% CO_2_ for 8 h. The cell viability and the cell number were determined before and after TrypLE incubation by staining 10 μL cell suspension with 1:1 (v/v) 0.4% Trypan Blue Solution (Gibco, USA). To eliminate dead cells, we followed the method of Dezawa et al. [27]. Briefly, the cells were centrifuged at 400 × *g* for 4 min at 20 °C and cells diluted in medium (10^6^ cells/5 mL) in a 15-mL centrifuge tube. The cell tube was vortexed for 3 min, then centrifuged at 740 × *g* for 15 min to remove the dead cells in the supernatant.

### Suspension culture of MSCs

A 96-well culture plate was coated with 50 µL polyHEMA/well (Sigma, USA) to prevent the cell attachment in the suspension culture. To prepare the coating solution, 1.2-g polyHEMA was dissolved in 40 mL of 95% ethanol then shaken at 37 ℃ for 8–10 h [27]. Coated plates were kept in sterilized, air-dried condition overnight at room temperature and were washed with PBS before use.

The semisolid medium was prepared by diluting methylcellulose (MC) (Sigma, USA) in StemMACS to a final concentration of 0.9%. About 8 × 10^3^ cells viable cells collected from stress condition were added in to 1 mL of semisolid medium and mixed thoroughly by pipetting. Then the mixture was transferred into polyHEMA pre-coated 96-well plates. Fresh StemMACS medium was added every three days. The cell clusters were formed and clusters larger than 50 μm in diameter were collected after 7–10 days. The cell viability of cell clusters was determined by staining with Trypan Blue dye.

### Immunofluorescence assay

To observe the expression of SSEA-3 by UCB-MSCs, cells were seeded on coverslips pre-coated with CELLStart until they reached 80% confluence. For cell cluster staining, the old medium was removed, and cell clusters were washed with PBS. Cells and clusters were fixed with 4% paraformaldehyde for 15 min and blocked with 5% FBS solution. The fixed samples were then incubated with FITC-conjugated SSEA-3 antibodies for 2 h at 25 °C in the dark. Cell nuclei were stained with DAPI (Invitrogen, USA). Cell images were captured by inverted microscope IX73 (Olympus, Tokyo, Japan) and analyzed using Image J software (version 1.46r).

### Growth factor analysis using Luminex assay

Growth factors such as hepatocyte growth factor (HGF), and vascular endothelial growth factor A (VEGF-A) were analyzed by Luminex assay using ProcartaPlexTM Multiplex Immunoassays (Human Custom ProcartaPlex 4-Plex Kit, Thermo Fisher, Massachusetts, US). UCB-MSC conditioned medium and thawed exosome suspension were kept on ice. Reagents and procedures were processed following the manufacturer's instructions. The growth factors luminescent signal was detected using Luminex™ 100/200™ system with xPONENT 3.1 software.

### Cellular senescence analysis of human primary UCB-MSCs

UCB-MSCs at passage 2, 3, 4 and 5 were analyzed for cellular senescence using Senescence Cells Histochemical Staining Kit (Sigma-Aldrich, Missouri, USA). The procedures were performed following to the manufacturer's instructions. UCB-MSCs were seeded in a six-well plate with a density of 4 × 10^5^ cells/cm^2^ and incubated for 48 h at 37 °C and 5% CO_2_ for cell attachment. After that, culture medium was removed, and cells were washed twice with 1X PBS before being fixed with 1X Fixation Buffer for 6 min at room temperature. Cells were then rinsed three times with 1X PBS and incubated in Staining Mixture overnight at 37 °C without CO_2_. The stained cells were washed twice with PBS before subjected to the second stain with DAPI Staining Solution (Abcam, Cambridge, UK) for 5 min at room temperature. After DAPI staining, cells were washed twice using PBS and imaged using an inverted microscope IX73 (Olympus, Tokyo, Japan). The images were captured and semi-qualitatively analyzed with Image J software (version 1.46r).

### Tube formation assay

UCB-MSC conditioned medium was prepared by culturing UCB-MSCs at 90% confluence in StemMACS with or without supplement for 48 h (CM-c and CM, respectively). Subsequently, the medium was collected and preserved at -80 °C for use in tube formation assay.

The assay was performed using an ab204726 Angiogenesis Assay Kit (In vitro) (Abcam, England). Briefly, extracellular matrix solution (Matrigel) was added into 96-wells plate and incubated for 1 h at 37 °C to allow the solution to form a gel. hUVECs were seeded at 1.5 × 10^4^ cells/well (3 replicates per group) on the gel in different media: EBM-2, CM-c, and CM. For the background control wells, no Matrigel was added. After 4 h and 8 h of incubation at 37 °C, the tube formation was examined using light inverted microscopy. The total tube length, total branching points and mean of tube length were analyzed by Wimasis software.

### Transwell co-culture system

SK-MEL cells were purchased from ATCC (American Type Culture Collection) (Manassas, VA, USA) and cultured in RPMI-1640 medium (Gibco, USA) supplemented with 10% FBS (Fetal bovine serum) (Gibco, USA), and 1% penicillin/Streptomycin (Gibco, USA), at 37 °C and 5% CO_2_. UCB-MSCs were harvested at passage four at approximately 70% confluence.

Cells were seeded on transwell plates (0.4 µm pore size) (Costar 3450; Corning, Inc., Corning, NY, USA) with UCB-MSCs in upper and SK-MEL cells in bottom wells. Cells were seeded at a density of 5 × 10^4^ cells/well in three types of culture medium: StemMACS, RPMI, and StemMACS mixed with RPMI at ratio of 1:1 (v/v) at 37 °C and 5% CO_2_. Along with the co-culture system, UCB-MSCs and SK-MEL cells were cultured as single cell type in the upper and bottom well, respectively, corresponding to the co-culture one. After 6 days of co-culture, cells were harvested and counted to determine the cell number.

### Reverse transcription polymerase chain reaction (RT-PCR)

Total RNA was isolated using TriSure kit (Bioline, USA), and DNA was removed by RNase-free DNase I (Thermo scientific, USA) for 30–60 min, at 37 °C. Collected mRNAs were used to synthesize cDNA using M-MLV Reverse Transcriptase (Enzynomics; Daejeon, Korea). cDNA fragments were amplified for three stemness genes *Oct3̸4*, *Nanog*, and *cMyc* on a PCR thermocycler (Eppendorf, Hamburg, Germany). The PCR products were run on a 1.2% agarose gel. The respective sequences of primers used for amplification are as follows: for Oct3/4 (5′-GACAGGGGGAGGGGAGGAGCTAGG-3′ and 5′-CTTCCCTCCAACCAGTTGCCCCAAA-3′), for Nanog (5′-CAAAGGCAAACAACCCACTT-3′ and 5′-TGCGTCACACCATTGCTATT-3′), and for cMyc (5′-GCGTCCTGGGAAGGGAGATCCGGAGC-3′ and 5′- TTGAGGGGCATCGTCGCGGGAGGCTG-3′).

### qPCR for differentiation genes

UCB-MSCs (*n* = 3) at passage 5 were cultured in the following media for 14 days in duplicates: StemPro™ Osteogenesis Differentiation Kit, StemPro® Adipogenesis Differentiation Kit, StemPro™ Chondrogenesis Differentiation Kit (Gibco, USA) to differentiate cells into the osteogenic, adipogenic and chondrogenic lineages, respectively. Cells of the same passage were used as undifferentiated controls. Cells were then lysed in TRIzol™ Reagent (Invitrogen/Thermo Fisher Scientific, USA) and total RNA was isolated according to the manufacturer’s instructions. Reverse transcription was carried out using a SuperScript™ IV First-Strand Synthesis System (Invitrogen/Thermo Fisher Scientific, USA); and qPCR was performed using PerfeCTa SYBR Green SuperMix (Quantabio, USA) according to the manufacturer’s instructions on a 7500 Real-Time PCR System (Applied Biosystems, Thermo Fisher Scientific, USA). The expression of the following genes was analyzed: alkaline phosphatase (ALP), parathyroid hormone-related protein (PTH-R), collagen type I (COL I) for osteogenesis, peroxisome proliferator-activated receptor gamma (PPARγ) and Leptin for adipogenesis, SOX9 and collagen type II (COL II) for chondrogenesis and GAPDH was used as a reference gene. Primer sequences are presented in Table 1.

**Table 1:** Primer sequences

Gene	Lineage	Primer	Primer sequence
ALP	Osteocyte	Forward primer	CCCGTGGCAACTCTATCTT
ALP	Osteocyte	Reverse primer	AGTCCACCATGGAGACATTC
PTH-R	Osteocyte	Forward primer	TGAACGGGAGGTGTTTGA
PTH-R	Osteocyte	Reverse primer	GGTGCATGTGGATGTAGTTG
COL I	Osteocyte	Forward primer	CCTGTCTGCTTCCTGTAAACTC
COL I	Osteocyte	Reverse primer	GTTCAGTTTGGGTTGCTTGTC
PPARg	Adipocyte	Forward primer	CCCTTCACTACTGTTGACTTCTC
PPARg	Adipocyte	Reverse primer	TGCAGGCTCCACTTTGATT
Leptin	Adipocyte	Forward primer	CCAGGATCAATGACATTTCACAC
Leptin	Adipocyte	Reverse primer	CATACTGGTGAGGATCTGTTGG
Sox9	Chondrocyte	Forward primer	AACAAGCCGCACGTCAA
Sox9	Chondrocyte	Reverse primer	TCTCGCTCTCGTTCAGAAGT
COL II	Osteocyte	Forward primer	ACACTCAAGTCCCTCAACAA
COL II	Osteocyte	Reverse primer	GTCAATCCAGTAGTCTCCACTC
GAPDH	Housekeeping genes	Forward primer	GGTGTGAACCATGAGAAGTATGA
GAPDH	Housekeeping genes	Reverse primer	GAGTCCTTCCACGATACCAAAG

### Assays for cell cycle and apoptosis analysis

Cell cycle was determined by using propidium iodide (PI) DNA staining. Briefly, 2 × 10^6^ cells were harvested and fixed in 70% (v/v) cold ethanol for 1 h. After washing 3 times with cold PBS, the supernatant was removed, and cells were resuspended in 1 mL of DNA staining solution (PBS containing 2% PI (wt/v) and 2% DNase free RNase (wt/v) for 30 min at room temperature and in the dark. Cells were then analyzed by FACS Canto II (BD, USA) using 488-nm laser for excitation. Red fluorescence of PI (> 600 nm) was measured with side scatter (SSC) and forward scatter (FSC), collecting at least 20,000 events. Residual debris was gated-out by using multilabel microbeads with sizes of 2 and 3 µm.

Apoptotic cells are detected by Annexin V-FITC and PI staining kit (BD, USA). Single and co-culture cells were collected and stained with Annexin V-FITC for 10 min, then with PI for 5 min at room temperature, in the dark. Stained cells were measured on FACS Canto II and data was analyzed by FACS Diva software. The results were presented as percentage of late and early apoptotic cells.

### Statistical analysis

All statistical analyses were performed using R software version 3.4.4. The statistically significant differences between groups and were assessed by *T* Test and Two-Way ANOVA and Tukey HSD tests. *p* < 0.05 was considered as statistically significant. All data were shown as means ± SD.

## Results

### UCB-MSC isolation and expansion

For UCB-MSC isolation, a total of 10 CB units were obtained for this study, and all CB samples were processed within 2 h after collection. The mean volume for blood units collected was 115 ± 23.27 mL and the average number of MNC collected was 2.2 ± 1.1 × 10^8^ cells.

**Fig. 1:**
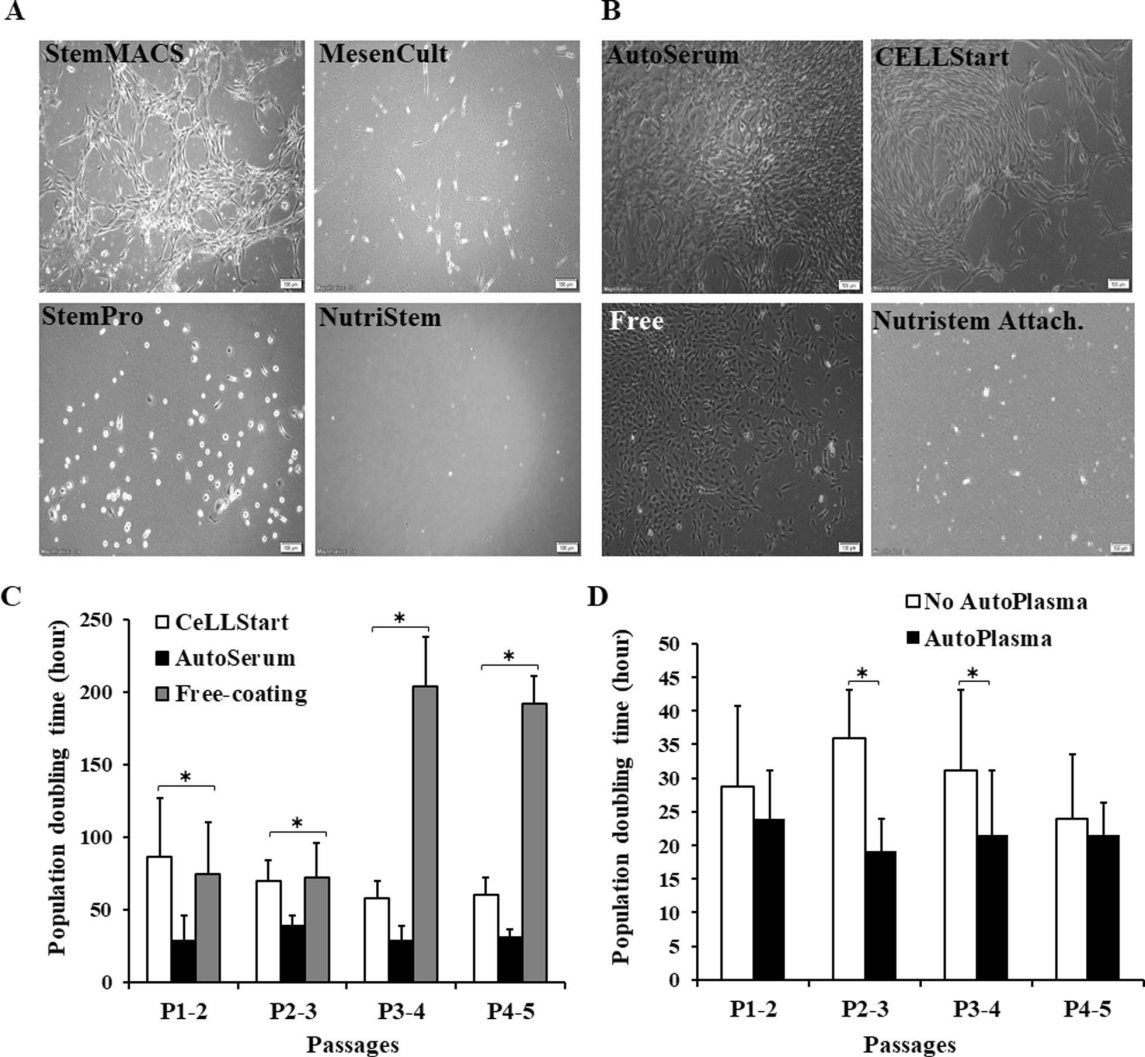
Isolated UCB-MSCs culturing with different commercial media and surface coating conditions. **a** Cells culturing with StemMACS, MesenCult, StemPro, and NutriStem. CFU can only be observed in 9 of 10 CB units culturing with StemMACS. **b** Cells culturing with StemMACS on the culture surface coated with AutoSerum, CELLStart, Free-coating, and Nutristem Attach. **c** Population doubling time (PDT) of MSC-like cells culturing in StemMACS with different surface coating: CELLStart, AutoSerum, and Free. **d**. Doubling time of MSC-like cells culturing in StemMACS supplement with or without 10% AutoPlasma in AutoSerum pre-coated plates. Bar = 100 µm, *p < 0.05

After isolation, MNCs were seeded into four commercial media to evaluate the isolation efficacy and expansion of MSCs: StemMACS, MesenCult, StemPro, and NutriStem. Together with the different media, four coating conditions were used: CELLStart, Nutristem Attach., AutoSerum and free-coated (Free). Adherent cells began to appear at the day 2nd post-seeding (POS) in StemPro medium, while in StemMACS and MesenCult media, the adhesion of cells appeared later, i.e. at day 6 and day 7 POS, respectively. There were only several cells that adhered in NutriStem media (Fig. 1a). MSC-like cells, which have spindle-shaped fibroblastic morphology, were first observed on day 5 POS in StemMACS plates coated with AutoSerum (Fig. 1a), and then in the other coating conditions, except Nutristem Attach. (Table 2, Fig. 1b). Meanwhile, there was no presence of MSC-like cells in the remaining plates (Fig. 1a) (Table 2).

**Table 2:** Detection of MSC-like cells in different commercial culture media and coating conditions

	Culture media							
	StemMACS		MesenCult		StemPro		MSC NutriStem	
Coating conditions	(+)	(−)	(+)	(−)	(+)	(−)	(+)	(−)
Free	+	+	−	−	−	−	−	−
CELLStart	+	+	−	−	−	−	−	−
Nutristem Attach	−	−	−	−	−	−	−	−
AutoSerum	+	+	−	−	−	−	−	−
	(+): with 10% AutoPlasma				(−): without 10% AutoPlasma			

Interestingly, MSC-like cells were detected in 9 out of 10 CB unit-derived MNCs cultured with StemMACS medium in three coating conditions: Free, CELLStart and AutoSerum, corresponding to an isolation efficacy of 90%. Most of MSC-like cells appeared as clusters or colonies. We then counted the number of MSC-like cell colonies (MCC) in each culture condition for each CB unit. The mean number of colonies per 10^8^ MNC in StemMACS was 1.56 ± 1.19, with the highest number of 2.08 ± 2.5 colonies in AutoSerum coating (Table 3). The time of colony detection varied from day 5 POS to day 26 POS (Table 2). In addition, the proliferation of MSC-like cells was induced by AutoSerum coating. We found that the population doubling time of cells was significantly shorter in AutoSerum coating compared to CELLStart and Free coating from passage 2–5 (*p* < 0.05) (Fig. 1c). We further tested if AutoPlasma could induce the proliferation of MSC-like cells by adding 10% autologous plasma into StemMACS medium in AutoSerum coating condition. The results showed that the doubling time of cells cultured with AutoPlasma supplement was significantly decreased from passage 2 to 4 (*p* < 0.05) (Fig. 1d), while this supplement had no effect on MSC isolation (Table 3).

**Table 3:** The number and day that MSC-like cell colonies (MCC) appeared in different coating conditions

Coating conditions	Isolated MCC per 10^8^ MNCs		MCC detection (Day post MNC seeding)	
	(+)	(−)	(+)	(−)
Free	0.23 ± 0.32	0.27 ± 0.2	14.5 ± 5.4	17.2 ± 8.2
CELLStart	0.48 ± 0.78	0.41 ± 0.62	12.3 ± 3.2	11.4 ± 6.2
Nutristem Attach	0	0	0	0
AutoSerum	2.08 ± 2.50	1.8 ± 1.9	10.8 ± 5.8	11.9 ± 6.9
	(+): with 10% AutoPlasma		(−): without 10% AutoPlasma	

These results indicated that MSCs present in the cord blood could be expanded in StemMACS culture medium with different coating conditions. The growth of isolated cells was induced by autologous plasma supplement at early passages.

### UCB-MSC characterization

To confirm that the isolated cells from umbilical cord blood were MSCs, we analyzed the CFU-F efficacy, immunophenotype, and differentiation ability of them. Since any MSC-like cells were detected only in StemMACS medium, we thus performed follow-up experiments with such cells again cultured in StemMACS with three coating conditions of CELLStart, AuroSerum, and Free.

**Fig. 2:**
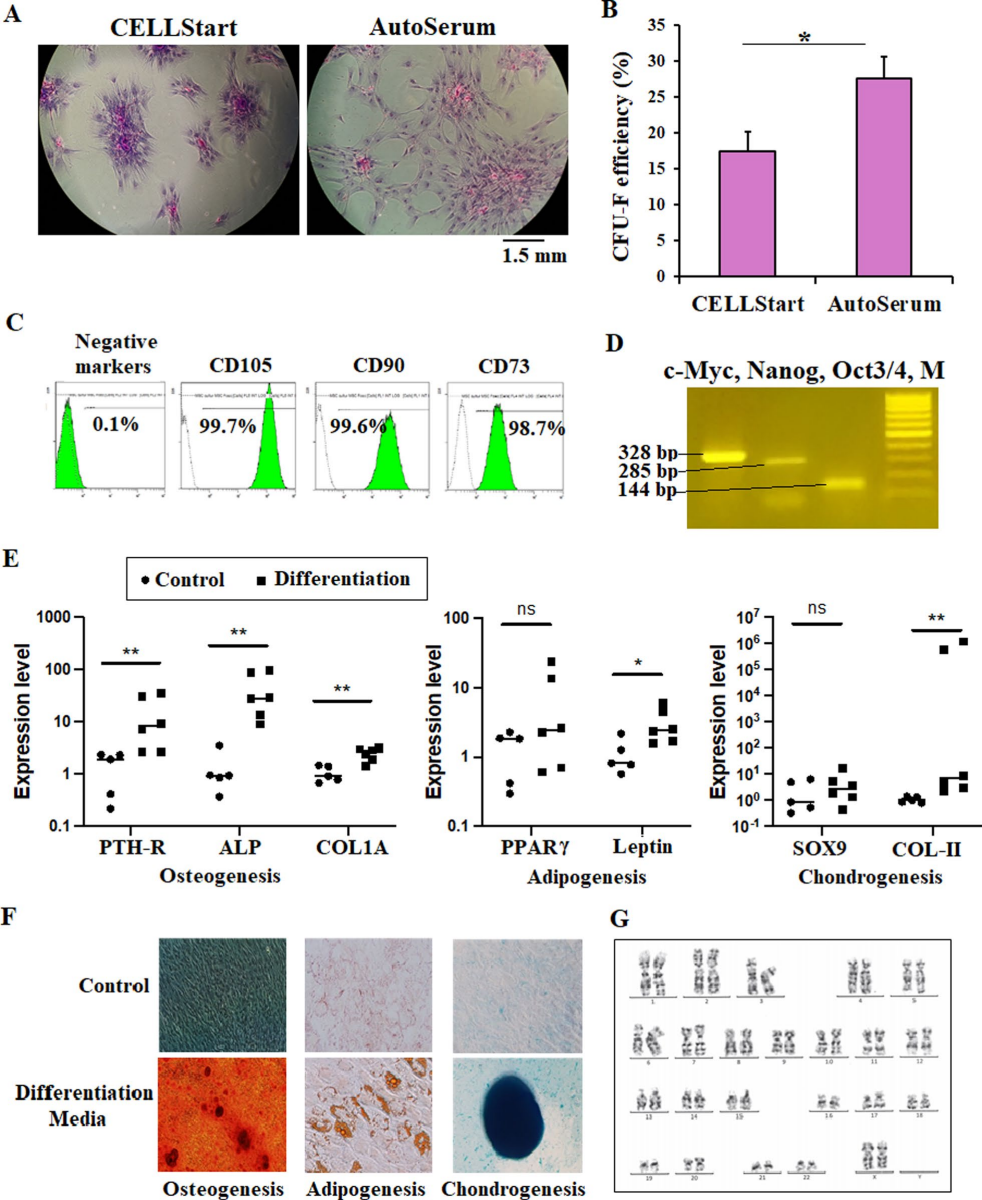
UCB-MSC characterization. **a** The morphology of CFU formed from cells cultured with StemMACS and the culture flask surface was coated with CELLStart and AutoSerum. **b** CFU efficacy in two coating conditions, CELLStart vs. AutoSerum. **c** Positive expression of MSC markers (CD90, CD73 and CD105) and negative markers (CD45, CD34, CD11b, CD19 and HLA-DR) from isolated UCB-MSCs. **d** RT-PCR revealed the expression of pluripotent markers of cMyc (328 bp), Nanog (285 bp) and Oct ¾ (144 bp), marker (M) 100 bp. **e**, **f** Trilineage differentiation of isolated UCB-MSCs. MSCs were cultured in osteogenic, adipogenic and chondrogenic induction media. Cells were used to performed qPCR (**e**) or stained with specific staining solutions (**f**) on day 14 and compared to undifferentiated control samples. **g** Karyotype analysis of UCB-MSCs showed no numerical or structural chromosome abnormalities. *p < 0.05; **p < 0.01

CFU-F assays were performed after 2 weeks of culture with a seeding density of 100 cells/cm^2^. Cell clusters were stained with Giemsa and the number of CFUs was counted. The results showed that MSCs were unable to form colonies without coating support (Free) but were able to form the colonies in the coating conditions (Fig. 2a). In CELLStart condition, the CFUs were looser and larger with lower number than that in AutoSerum coating (Fig. 2a). Furthermore, the data also pointed out a clear difference in CFU formation with a significantly higher efficacy in AutoSerum coating (27.5%) compared to CELLStart coating (17.4%) (*p* < 0.05) (Fig. 2b), which proved the efficiency of CFU forming capacity supported by using autologous serum coating.

The results of immunophenotyping indicated that isolated cells cultured with StemMACS supplemented with or without 10% AutoPlasma exhibited MSC surface makers of which 99.84 ± 0.11% for CD90, 97.07 ± 1.88% for CD73 and 99.79 ± 0.16% for CD105. Negative markers such as CD90, CD45, CD34, CD11b, CD19 and HLA-DR were 0.23 ± 0.11% (Fig. 2c). These cells also expressed Nanog, Oct3/4 and cMyc (Fig. 2d). We examined the trilineage differentiation ability of these cells in vitro. After 14 days, osteogenic-induced cells showed osteogenic phenotypes by expressing significantly different levels of PTH-R, ALP, and COL1A (*p* < 0.01) compared to the control. Likewise, adipogenic and chondrogenic-induced cells expressed Leptin and COL-II, respectively (*p* < 0.05). Of note, there were higher expressions of PPARγ in the adipogenic and SOX9 in chondrogenic–induced cells compared to undifferentiated control; however, it was not significant (Fig. 2e). Nevertheless, calcium deposits assessed by Alizarin Red staining were positive in osteogenic-induced cells compared to that in the control (Fig. 2f). Lipid-rich vacuoles were observed in adipogenic-induced cells, which are confirmed by Oil Red O visualization (Fig. 2f). In chondrogenic induction, we observed the aggregation of cells into cartilage-like structures. These aggregates were positive when staining with Alizarin Blue (Fig. 2f). The karyotyping test was performed on UCB-MSCs at passage 5. The results showed no numerical (euploid and aneuploid) or structural chromosome abnormalities, illustrating the stability of chromosomes during culture period (Fig. 2g).

**Fig. 3:**
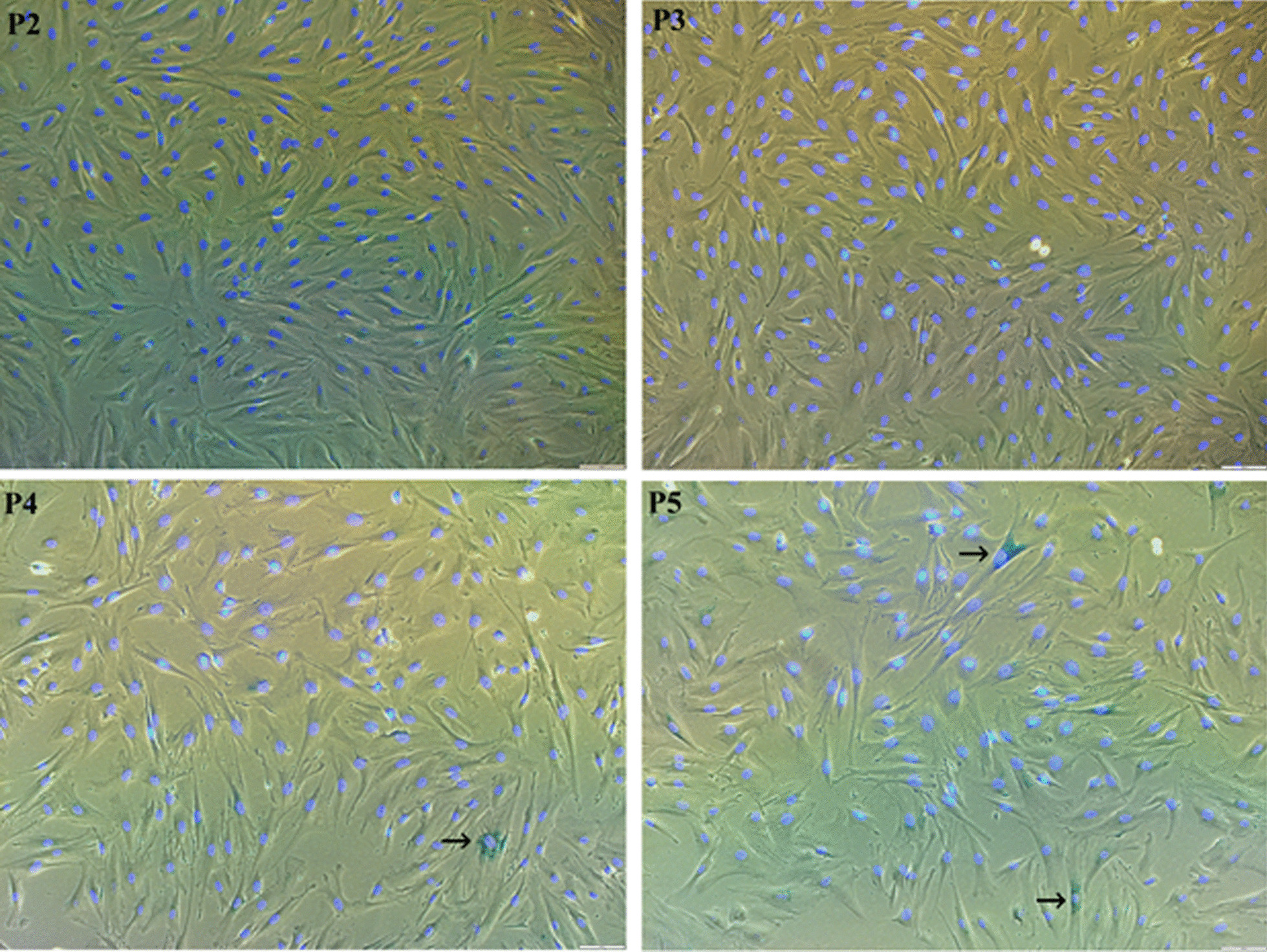
Cellular senescence in UCB-MSC population at passage 2 (P2), 3 (P3), 4 (P4), and 5 (P5). The cells positive with *β*-galactosidase activity were indicated by arrows. Bar = 50 µm

We also tested the cellular senescence of UCB-MSCs at different passages. As shown in Fig. 2, there were only few cells that expressed the blue colour after staining with Senescence Cells Histochemical Staining Kit (Sigma-Aldrich) (Fig. 3, arrows). The percentages (%) of *β*-galactosidase activity positive cells in UCB-MSC populations were 0.044 ± 0.107% (passage 2), 0.118 ± 0.147% (passage 3), 0.137 ± 0.166% (passage 4), and 0.245 ± 0.230% (passage 5). Data demonstrated that the cellular senescence occupied a very small number among UCB-MSC population, and the number of senescent cells tended to increase by passages but were not significantly different (*p* < 0.05).

In summary, these results proved that MSC-like cells isolated by the method described in this study exhibited all characteristics that meet the requirement of ISCT standards for MSC cells. This indicates the isolated cells were indeed MSCs.

### Muse cells in UCB-MSC population

**Fig. 4:**
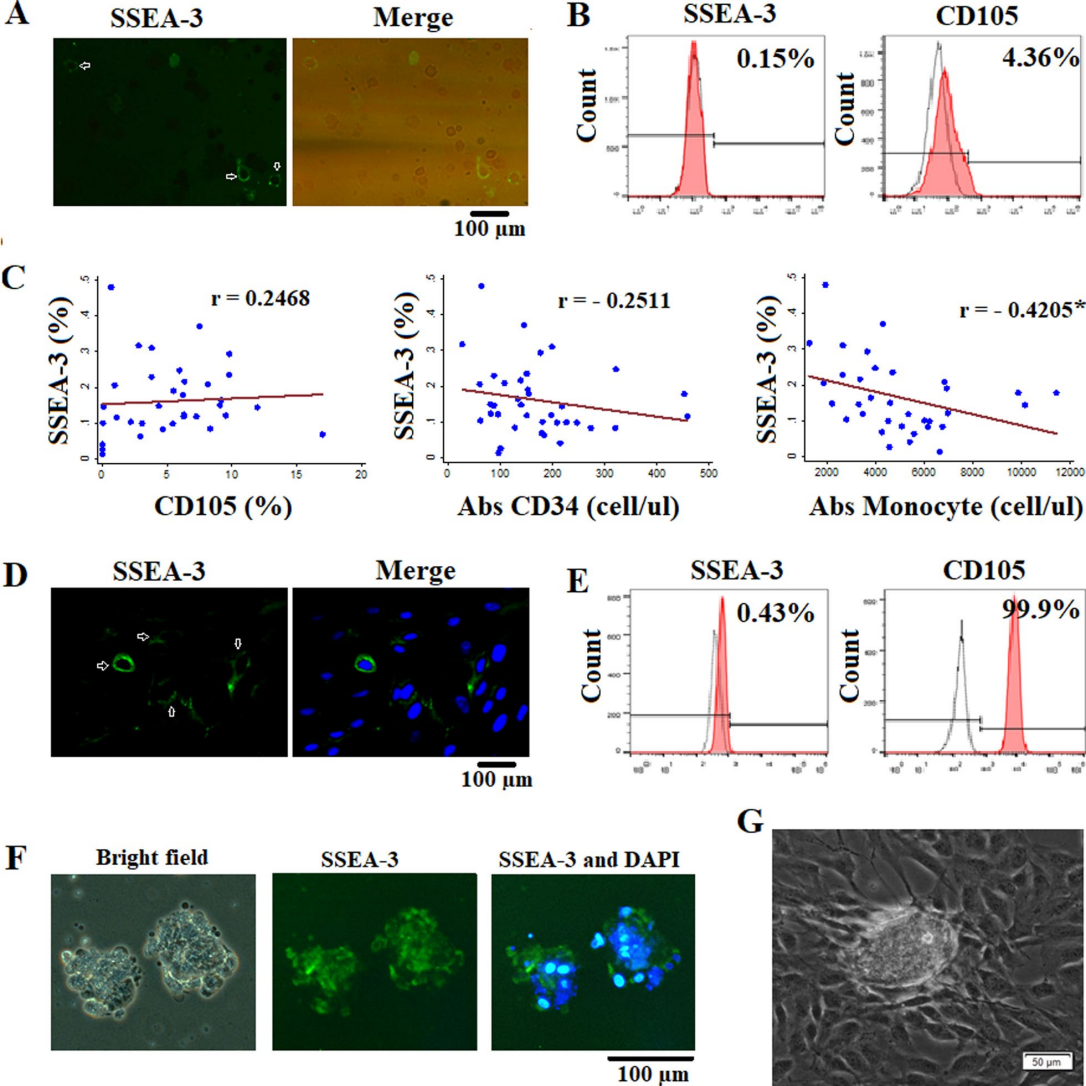
Muse cell identification in cord blood MNC and MSC populations. **a** Cells were positive with SSEA-3 marker in UCB-MNC population. **b** Percentage (%) of SSEA-3^+^ cells and CD105^+^ cells among UCB-MNCs. **c** The correlation of the rate of SSEA-3^+^ cells with the percentage (%) of CD105^+^ cells, the density of CD34^+^ cells, and the density of monocytes among UCB-MNCs. **d** Cells were positive with SSEA-3 marker in UCB-MSC population. **e** Percentage (%) of SSEA-3^+^ cells and CD105^+^ cells among UCB-MSCs. **f** Isolated Muse cells grew as embryonic bodies in semisolid condition. **g** Expanded Muse cells grew as cluster in adherent condition

In recent 10 years, a number of studies have reported about the existance of a small populations of cells within MSC populations which express pluripotent markers and are called Muse cells (Multilineage-differentiating stress-enduring cells). Therefore, we checked whether UCB-MNCs and MSCs isolated by our method contained these cells or not. Results showed that there was a population of SSEA-3^+^ cells present in our MNC populations that could be confirmed by immunofluorescence imaging (Fig. 4a). Additionally, data from flow cytometry showed that in 35 cord blood MNC samples, the SSEA-3^+^ cells and CD105^+^ cells accounted for 0.18 ± 0.09% and 5.9 ± 2.8%, respectively (Fig. 4b). The percentage of cells which expressed both markers was 0.07 ± 0.03%. We further analyzed the relationship between the absolute number of CD34^+^ cells and monocytes to the percentage of SSEA-3^+^ cells among UCB-MNCs. Interestingly, there was an inverse correlation between these cell populations with *r*-spearman values of − 0.2511 (SSEA-3^+^ vs. CD34^+^) and − 0.4205* (monocytes vs. SSEA-3 + , *: *p* < 0.05). Meanwhile, the rate of SSEA-3^+^ cells showed a positive correlation with CD105^+^ cells (*r*-spearman = 0.2468) (Fig. 4c).

The presence of Muse cells was also observed in UCB-MSC populations (Fig. 4d). Through flow cytometry, we calculated the rate of these cells was 0.3 ± 0.1% (Fig. 4e). In order to enrich Muse cells, we perfomed the stress-induced condition by long-time treated MSCs with trypsin–EDTA for 8 h at 37 °C. The viability of cells remaining in digestic enzyme-stress culture was 6.3 ± 4.52%. These cells were continued to be cultured in semisolid medium MC in culture dishes pre-coated with polyHeMA. In this culture condition, stressed cells were grown as clusters and expressed SSEA-3 (Fig. 4f). The efficacy of embryonic body forming ability in the Muse cell population was 0.02 ± 0.09% with a mean size of the embryonic bodies of 98.4 ± 4.3 (µm). Moreover, when culturing these stressed cells in adherent conditions, there were also cell clusters among the other adherent cells indicating the character of Muse cells (Fig. 4g).

These results indicated that there were Muse cells in the UCB-MSC population isolated by our method and these cells were able to survive digestic enzyme-stressed conditions.

### UCB-MSC-conditioned medium induced angiogenesis processes in vitro

**Fig. 5:**
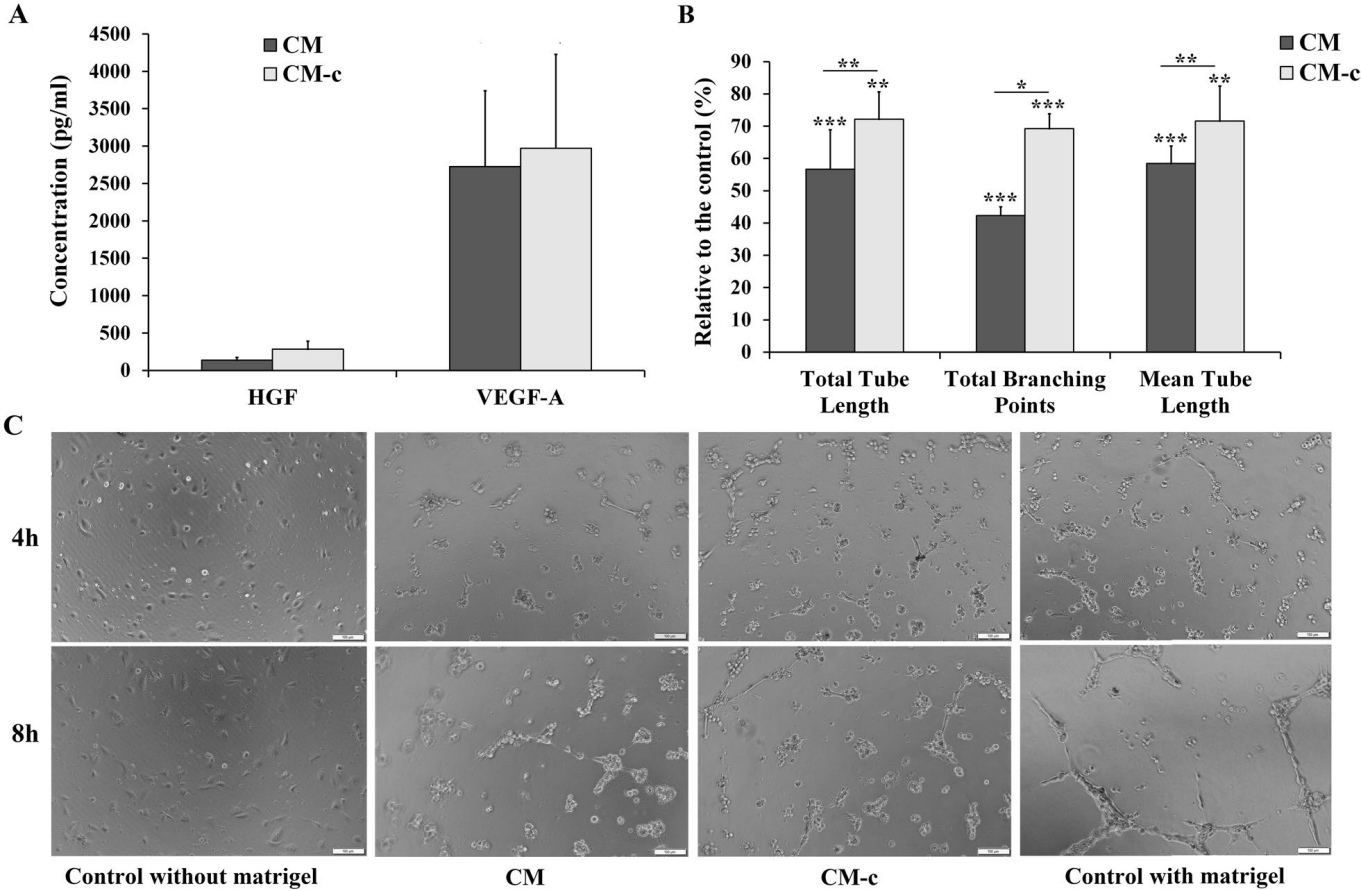
UCB-MSC conditioned medium induced the tube formation of hUVECs. **a** Expression of VEGF-A and HGF in UCB-MSC conditioned medium with (CM-c) or without (CM) supplement was determined by a bead-based immunoassay. **b** Quantitavie analysis of tube formation of hUVECs in CM and CM-c. **c** The images of tube-structure was observed by inverted microscope. Bar = 100 µm. Data were presented as mean ± SD, with *n* = 3. (* *p* < 0.05, ** *p*-value < 0.01, *** *p*-value < 0.001)

To examine whether the secretome in UCB-MSC conditioned medium can accelerate angiogenesis in vitro, a tube formation assay was performed. Of note, the supplement for MSC culture medium in StemMACS kit consisted of growth factors which could affect to the tube formation efficacy. Therefore, the effects of culture media with and without supplement on the hUVECs-derived tube formation assay were needed to be taken into account. Herein, hUVECs were cultured in 4 conditions: completed EBM-2 medium with Matrigel (control), completed EBM-2 medium without Matrigel (control without Matrigel), StemMACS conditioned medium with supplement (CM-c), and StemMACS conditioned medium without supplement (CM). The presence of VEGF-A and HGF, two main regulators for angiogenesis, was determined in both CM and CM-c (Fig. 5a). Interestingly, the concentrations of VEGF-A in both CM and CM-c were rather high with 2726.2 ± 1012.8 and 2970.9 ± 1257.4 (pg/mL), respectively. Similarly, the amount of HGF was also high in both CM and CM-c with concentrations of 136.4 ± 38.4 and 282.3 ± 107.5 (pg/mL). The concentrations of these factors in CM were not significantly different compared to that of CM-c (*p* > 0.05). It is worth to note that the CM was not supplemeted with growth factors at the beginning of UCB-MSC culture. That mean UCB-MSCs secreted the angiogensis regulators into their culture medium. These regulators play a role in inducing tube formation in vitro when culturing hUVECs on Matrigel (Fig. 5b). At 8 h, the relative total tube length in CM and CM-c compared to the control were 56.7% and 72.1%, respectively. However, the mean tube length and the total branching points were smaller in CM and CM-c than in the control (*p* < 0.05) (Fig. 5b). We observed the appearance of small tubes after 4 h of culturing hUVECs in CM and CM-c (Fig. 5c). Taken together, these data suggest that the conditioned medium of UCB-MSC contained the angiogenesis factors and promoted the angiogenesis in vitro.

### UCB-MSC inhibited the growth of SK-MEL cancer cells in co-culture system

**Fig. 6:**
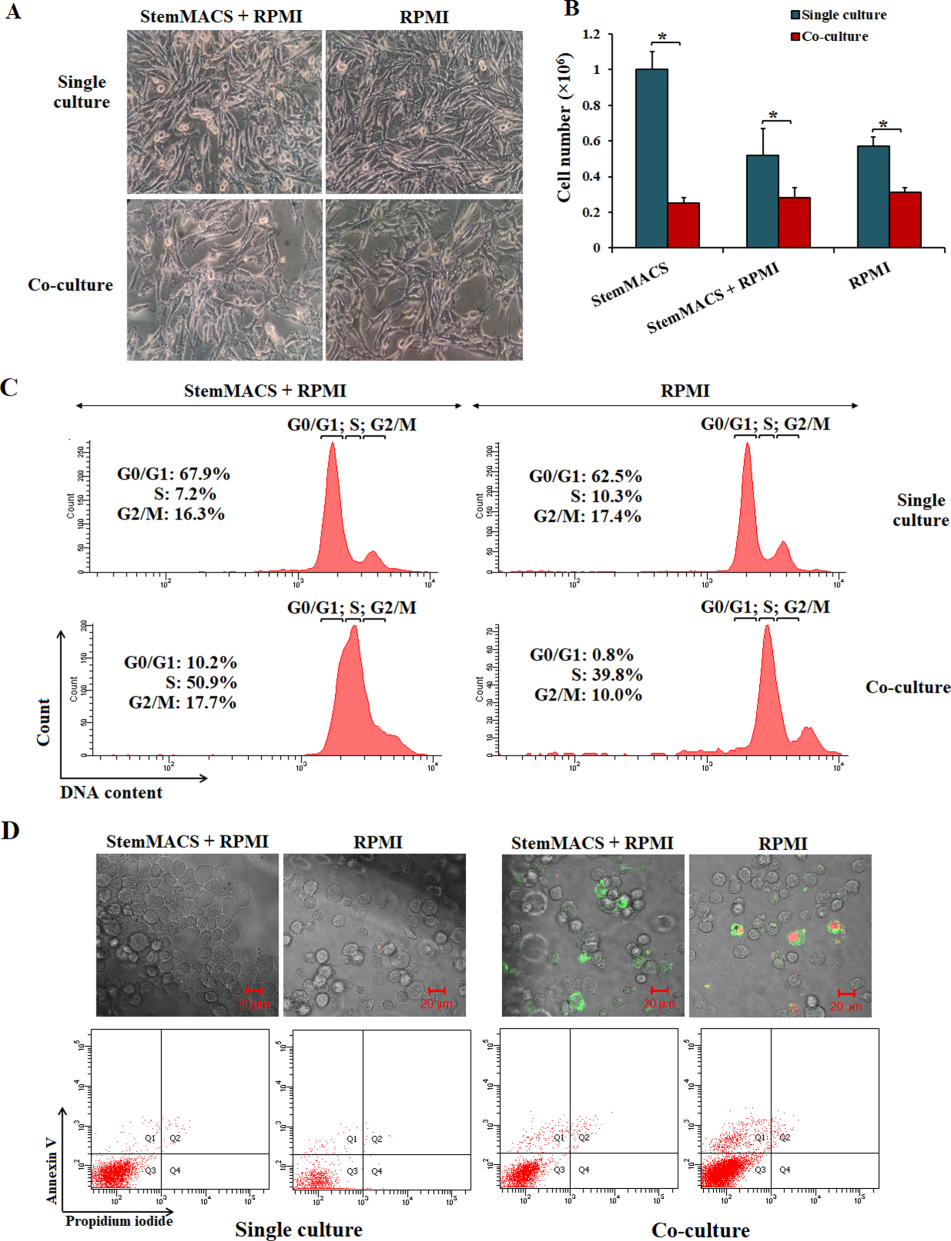
UCB-MSCs inhibited the growth of SK-MEL cancer cells in co-culture system. **a** Morphology of SK-MEL cells in single and co-culutre in two different culture media. **b** The number of SK-MEL cells decreased when co-culturing with UCB-MSCs in all three types of culture media used in the experiment. **c** The cell cycle of SK-MEL cells was inhibited at S phase after 6 days of co-culturing with UCB-MSCs. **d** The apoptosis SK-MEL cells were revealed by immunoflouresence images by staining with Annexin V-FITC (green) and PI (red). *p < 0.05

SK-MEL cancer cells were co-cultured with UCB-MSCs in 6-well transwell plates, with cancer cells in the bottom and UCB-MSCs in the upper wells. In order to be sure about the effect of only UCB-MSCs on cancer cells but not medium culture factors, we performed several types of medium arrangements: completed StemMACS (StemMACS), completed RPMI (RPMI), and completed StemMASC mixed with completed RPMI at a ratio (v/v) of 1:1 (StemMACS + RPMI). In all three types of culture medium, the number of SK-MEL cells was decreased after 6 days of co-culture (Fig. 6a), with a growth of cells inhibited by more than 50% in StemMACS + RPMI and RPMI only (Fig. 6b). Interestingly, the inhibition was higher in StemMACS with the number of cancer cells decreased 4 times (Fig. 6b). Flow cytometry analysis revealed that the cell cycle of SK-MEL cells in co-culture was arrested at S phase with the percentages of the cells in this phase increased from 8.5 ± 1.1% and 9.3 ± 0.6% to 49.7 ± 2.1% and 39.6 ± 1.4%, in StemMACS + RPMI and RPMI, respectively (Fig. 6c). Moreover, we noticed that apoptotic cells increased in both co-cultures compared to the single culture, with the average percentage of apoptosis of more than 10% compared to less than 2%, respectively (Fig. 6d).

**Fig. 7:**
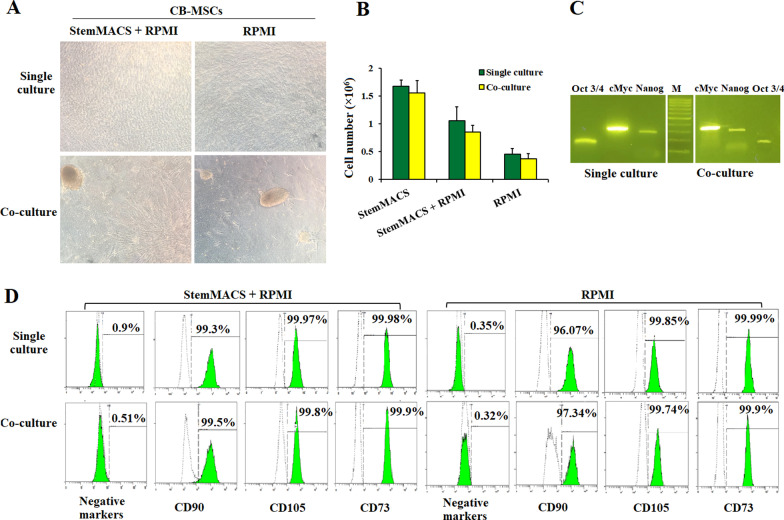
UCB-MSCs maintained their characteristics in the cancer cell co-culture condition. **a** The morphology of UCB-MSCs growth as monolayer in single culture, and as colonies in co-culture. **b** There was no significant different in the number of UCB-MSCs between single and co-culture system. **c** The expression of three stemness genes *cMyc*, *Oct3/4*, and *Nanog* of UCB-MSCs in two culture conditions. **d** The expression of MSC positive and negative markers in single and co-culture systems

We also checked the effect of co-culture on the growth of UCB-MSCs. As shown in Fig. 7, co-culture with SK-MEL cells induced changes in the morphology of cells but did not affect the number of UCB-MSCs. In single culture, UCB-MSCs grew as monolayer, meanwhile in co-culture most of cells clustered in colonies (Fig. 7a). The number of cells in both types of culture was not different between the single and co-culture, but the total number of UCB-MSCs was highest in StemMACS, followed by StemMACS mixed with RPMI and lowest in RPMI alone (Fig. 7b). This could be explained since StemMACS is the optimized medium for MSC culture. For stemness gene expression and MSC markers, there was no significant difference between the single and co-culture of UCB-MSC (*p* > 0.05) (Fig. 7c, d).

## Discussion

### Efficient and high-yield isolation and culture of MSCs from human umbilical cord blood

In this study, we aimed to select a GMP-standard culture condition for UCB-MSC isolation and expansion by using different xeno-free and serum-free commercial media products. Ten full-term cord blood units were collected and met the minimum criteria described by Bieback and Netsch [28] about blood volume collected, storage time, and number of isolated MNCs. Surprisingly, among four different MSC culture kits (StemMACS, MesenCult, StemPro, and NutriStem), only StemMACS showed a suitable ability for the isolation of MSCs from umbilical cord blood with a substantially higher success rate of 90% (9/10 samples). In a study of Schubert et al., they got similar results that equine adipose MSCs could not grow in StemPro® MSC SFM CTS ™ but did grow in StemMACS medium [29]. The yield of MSC isolation in our study was rather high compared to that of previous studies [14–16]. The amount of MSC-like cell colonies (MCC) obtained was 1.56 ± 1.19 MCC/10^8^ MNC, which is higher compared to the results published by Javed et al. [17], with MCC numbers obtained from full-term units (37–40 weeks of age) was 0.02 ± 0.02 MCC/10^8^ MNC, and who used EGM-2 medium (cEGM2) supplemented with FBS for isolation. Another research published in 2011 by Zhang et al. using Dulbecco’s Modified Eagle’s Medium, claimed that they successfully obtained MCCs at 2.10 ± 1.93 MCC/10^8^ MNC, following a low isolation success rate of is only 47.8% [28], which is lower than our results (90%).

The characteristics of UCB-MSCs after isolation and culture were determined. Our UCB-MSCs satisfied the criteria of the minimal standards for MSCs proposed by the International Society for Cellular Therapy (2006) [1], with suitable adhesion ability, high expression of MSC positive markers (> 97%) and lack of negative markers (< 1%). Moreover, these UCB-MSCs also expressed three key transcription regulatory genes for pluripotent stem cells, such as *Nanog*, *Oct3̸4*, and *cMyc*. Particularly, the expression of cMyc was high in UCB-MSCs, which may explain to the high proliferation rate of the cells that closely resembles that of embryonic stem cells. This result was consistence with the study of Jin et al. when comparing BM-MSCs, AT-MSCs and UCB-MSCs, and they demonstrated that UCB-MSCs had the highest proliferation rate among three types of cells [5, 8]. We also confirmed in vitro osteogenic, adipogenic, and chondrogenic differentiation capability of our UCB-MSCs by gene expression levels and functional indicators. In addition, we ensured that the entire culture process did not cause any significant damage for the cells’ genome by performing karyotyping analysis, which confirmed that there were no numerical or structural chromosome abnormalities in cultured UCB-MSCs.

Taken together, these results demonstrated that we were successful in the isolation of MSCs from cord blood with a high efficiency of 90% when using the StemMACS medium kit.

Previous studies reported that the isolation success rate of MSCs depends on cell adhesion and interaction with culture surface. In this study, along with StemMACS, we used four different coating culture surface conditions with CELLStart, Nutristem Attach., AutoSerum, and Free. MCCs appereared in all conditions except Nutristem Attach. It means that StemMACS itself could induce the adhesion of UCB-MSCs. This result was consistent with the instruction of StemMACS kit that suggested that it is not necessary to coat culture surfaces since the kit already contains factors that induce the adhension ability of MSCs [29]. Moreover, the results in our study indicated that the adhesion capability of UCB-MSCs at isolation stage depended on coating solutions, with negative results for NutriStem Attach., meanwhile CellStart and AutoSerum showed similarly positive effects on UCB-MSC isolation. However, the effect of these two coating conditions was different in terms of cell growth induction. Our results indicated that the PDTs of isolated MSCs were significantly changed (*p* < 0.05) from passage 1 to passage 5 in different coating conditions. The lowest PDT value was at AutoSerum coating (31.8 h), followed with CELLStart (68.4 h) and Free (135.6 h). The proliferating rate of UCB-MSCs was reported to widely range from about 30 h to hundred hours [8, 19, 30]. All these published data used FBS as supplement with the culture medium. Compared to them, our study showed the equivalent growth capacity of UCB-MSCs. Remarkably, the PDT values had a trend of decreasing in AutoSerum and CELLStart coating, but to increase in Free coating. UCB-MSCs grown in StemMACS without surface coating had the longest of doubling time (about 200 h) compared to CELLStart and AutoSerum coating. Moreover, there was no CFU-F formation observed in Free coating conditions. Of note, many publications had pointed out the function of coating components on MSC growth and survival [31–33]. Taken together, these results suggest that StemMACS may not provide suffcient factors for cell adhension during cell growth phase, and UCB-MSCs need surface coating support for more optimized growth.

In this study, we selected CELLStart as one of the coating factors since CELLStart™ is a defined substrate, containing only material of human origin. This product is cGMP manufactured and consists of a mixture of matrix proteins, which is an ideal surface for cells [34, 35]**.** However, in our study**,** compared to CellSart, AutoSerum coating resulted in even better effects on UCB-MSC growth, as well as on self-renewal capacity with smaller PDT values and higher CFU-F formation ability (*p* < 0.05). This coating solution even showed earlier time for MCC appearance than other coating conditions, though it was not significant (*p* > 0.05). The typical components found in AutoSerum are numerous and include a wide range of macromolecules, similar to FBS [2, 29]. Exactly which are the key components in this serum required to induce the proliferation of primary UCB-MSCs remains to be elucidated. Nevertheless, our study indicated that AutoSerum may benefit in the way of autologous use to which the cells can adapt very well. This advantage was confimed by the use of autologous plasma in cell culture. In fact, for the optimization of culture conditions, we used 10% of heat-inactivated autologous plasma (AutoPlasma) as a medium supplement for boosting the MSC growth rate. The efficiency of autologous serum has been shown in research conducted by T. Nazari-Shafti et al. in 2018, or Alexander Popov et al. in 2019, in which the presence of human serum in culture improved the proliferation capacity of UCB-MSCs [2, 36]. Furthermore, another study in 2019 of Thaweesapphithak et al. [25] indicated that human serum-supplemented media not only promoted better MSCs proliferation, but also had an equivalent immunosuppressive and differentation-supportive effect comparable to FBS, which is suitable for cell therapy in the future. Similar outcomes were also observed in our study, in which UCB-MSCs in medium supplemented with AutoPlasma showed a considerably higher proliferation rate.

Summarizing the results UCB-MSC isolation and expansion in our study, we demonstrated that StemMACS had a high efficiency for cell isolation. For optimal cell growth and self-renewal ability, it needs the support of coating agents, and autologous serum and plasma can improve cell proliferation, especially in early passages. Our isolation and culture method in compliance with good manufacturing practice (GMP) is essential for the development of UCB-MSC-based therapies.

### Functionalization of UCB-MSCs

In addition to the well characterization of UCB-MSCs, we also tested the utility of these cells for the purpose of further developing this type of MSCs for clinical applications. For about the last 10 years, there has been increasing evidence for the existence of a small population of multilineage differentiating stress enduring cells (Muse cells) among a variety of cell populations from different sources such as bone marrow (0.03%), BM-MSCs (1–2%) [37], AD-MSC (3.2%) [38], and UC-MSC (5%) [39]. These cells were defined as pluripotent stage-specific embryogenic antigen 3 (SSEA-3)^+^ mesenchymal stem cells, which can spontaneously differentiate into cells of the three germ layers both in vitro and in vivo [40]. Muse cells have various promising characteristics compared to other types of stem cells. They are highly resistant to homologous graft rejection, have the ability of homing toward damaged tissues, and they are considered non-tumorigenic, which means they do not form teratomas [40]. This marks Muse cells as a potential source for pluripotent stem cells [40, 41]. In this study, we determined the percentage of Muse cells (SSEA-3^+^CD105^+^) in UCB-MNC with 0.07%, which is lower than that in BM-MNC [40], in UCB-MSCs was 0.3%. Interestingly, the number of SSEA-3^+^ cells in UCB-MNCs had an inverse correlation with the number of monocytes, which is contrary to the relationship of the number of CD34^+^ cells and monocytes (*p* < 0.05). This could be used as an index for the cord blood selection in term of SSEA-3^+^ isolation. Besides, the enriched Muse cells in UCB-MSC population by long-term treatment with trypsin enzyme showed an ability in cluster formation positive for SSEA-3^+^ in both suspension and adherent culture, resembling the formation of embryonic and induced pluripotent stem cells. Even through the percentage of Muse cell population in UCB-MSCs was smaller than that what was reported in some other MSC resources, our results still revealed that UCB-MSCs could be a potential source for Muse cell isolation and expansion.

Currently, the interaction between MSCs and cancer cells is still unclear, whether MSCs inhibit or promote cancer growth is still a controversial question. Several studies suggested that UCB-MSCs appeared to affect pathways that may suppress both proliferation and apoptosis in cancer cells [42–44]. The dual role of MSCs can be described as a double-edged sword in cancer progression. Therefore, it is important to understand its dual role in cancer development. This study examined the indirect interaction between hUCB-MSCs and SK-MEL cells using a co-culture model in which the two cells are indirectly interacting through a 0.4 µm membrane filter. Using this system, we can check if the paracrine signaling of both cell types can affect each other. After 6 days of co-culture, the proliferation of SK-MEL was inhibited by 50–70% compared to that in the single culture. The decrease in the number of cancer cells may be caused by the cell cycle being arrested at S phase since the percentage of SK-MEL cells in this phase was significantly increased about 5 times after co-culture with UCB-MSCs. Research by Yu et al. reported about the "capture" induction at phase S of bladder cancer cells when co-culturing them with AD-MSCs [45]. In our study, the percentage of apoptosis cells also increased about 5 times in SK-MEL co-cultured with UCB-MSCs. This suggests that UCB-MSCs have the ability to inhibit the growth of melanoma cells, and to induce apoptosis. Interestingly, co-cultured UCB-MSCs still maintained their normal proliferation rates while fully expressing MSC-specific surface markers and a pluripotent gene expression profile. Of note, the morphology of UCB-MSCs changed with the formation of clusters in the co-culture wells while cells grew as monolayer in the single-culture. In a study of El-Badawy, they observed the same morphology of BM-MSCs when co-cultured with cancer cells in the same Transwell system [46]. Clearly, more experiments should be performed to elucidate the mechanism behind the morphology change of UCB-MSCs when co-culturing with cancer cells, as well as the effect of this stem cell type on cancer cells. Nevertheless, our initial results revealed that the proliferation of SK-MEL cells was inhibited by a co-culture with UCB-MSCs.

Besides the paracrine effect of UCB-MSC on cancer cells, we also checked if the secrotome of these cells could induce the angiogenesis process in vitro. VEGF-A is one of the most important regulators of angiogenesis [47]. Our study clearly showed that this factor was secreted by UCB-MSCs with high concentrations. Besides, the hepatocyte growth factor, another angiogenic stimulator, was expressed in UCB-MSC conditioned medium. Previous studies revealed that VEGF-A combined with HGF resulted in a potent angiogenic effect in a mouse model of limb ischemia [47]. The combination of these two molecules can promote neovascularization of endothelial cells [48]. This effect was also observed in our study when applying UCB-MSC conditioned medium on hUVECs. In both CM and CM-c groups, the tube started to form early, at 4 h of treatment. At 8 h, the total tube length in CM and CM-c reached a half and two third of the control, respectively. However, the mean length of tubes was smaller and no network was formed. Nonetheless, our results indicated that UCB-MSC CM, without supplement, had the ability to induce an angiogenesis process by stimulating the tube formation of hUVECs.

## Conclusion

In conclusion, this is the first study using xeno-free and serum-free medium to isolate and expand of MSCs from cord blood. By using four commercial kits, we showed that only the StemMASC kit had the ability to isolate MSCs successfully. For the optimization of cell proliferation and renewal capacity, autologous serum should be used as a coating factor and autologous plasma should be added in the culture medium at early passages. UCB-MSCs isolation by our method meets the standard requirements for MSC characteristics proposed by ISCT. Moreover, the cell population contained Muse cells, have the ability to induce tube formation of endothelial cells, and inhibition of melanoma SK-MEL cells. Certainly, more studies should be carried out to further elucidate the mechanisms of these effects, but even so, our study reveals that UCB-MSCs can be isolated and expanded under GMP standards and could be developed for cell-based therapeutic applications.

## Data Availability

The data that support the findings of this study are available upon request from the corresponding author.

## Abbreviations

AD: Adipose tissue
ALP: Alkaline phosphatase
ATCC: American type culture collection
AutoPlasma: Autologous plasma
AutoSerum: Autologous serum
BM: Bone marrow
CD: Cluster of differentiation
cDNA: Complementary DNA
CFU-F: Fibroblast colony-forming unit
CM: Conditioned medium
COL I: Collagen type I
COL II: Collagen type II
CPDs: Cumulative population doublings
CT: The time in culture between two time points
DNA: Deoxyribonucleic acid
EDTA: Ethylenediaminetetraacetic acid
FBS: Fetal bovine serum
FITC: Fluorescein isothiocyanate
GAPDH: Glyceraldehyde 3-phosphate dehydrogenase
HGF: Hepatocyte growth factor
HLA-DR: Human leukocyte antigen-DR isotype
HUVEC: Human umbilical vein endothelial cell
ISCN: An international system for human cytogenomic nomenclature
ISCT: The International Society for Cellular Therapy
MC: Methylcellulose
MCC: MSC-like cell colonies
MNC: Mononuclear cells
MSC: Mesenchymal stem cell
Muse: Multilineage-differentiating stress-enduring
PBS: Phosphate buffer saline
PDT: Population doubling time
PE: Phycoerythrin
PFA: Paraformaldehyde
PI: Propidium iodide
PPARγ: Peroxisome proliferator-activated receptor gamma
PTH-R: Parathyroid hormone-related protein
RNA: Ribonucleic acid
RT-PCR: Reverse transcription polymerase chain reaction
SSEA-3: Stage-specific mouse embryonic antigen
UCB: Umbilical cord blood
VEGF-A: Vascular endothelial growth factor A
